# Impact of busulfan exposure stratification on transplant outcomes and immune reconstitution in children with severe aplastic anemia

**DOI:** 10.3389/fphar.2026.1897156

**Published:** 2026-09-04

**Authors:** Yanxiao Li, Xiaoying Zhai, Qianqian Zhang, Yile Zhao, Chenhong Jia

**Affiliations:** 1 Department of Hematology and Oncology, Hebei Children’s Hospital (Hebei Provincial Clinical Research Center for Child Health and Disease), Shijiazhuang, Hebei, China; 2 Department of Pharmacy, Shijiazhuang Hospital of Traditional Chinese Medicine, Shijiazhuang, Hebei, China; 3 Department of Pharmacy, Hebei Children’s Hospital (Hebei Provincial Clinical Research Center for Child Health and Disease), Shijiazhuang, Hebei, China

**Keywords:** allogeneic hematopoietic stem cell transplantation, busulfan, children, immune reconstitution, pharmacokinetics, severe aplastic anemia, therapeutic drug monitoring

## Abstract

**Background:**

Severe aplastic anemia (SAA) is a life-threatening bone marrow failure disorder. Allogeneic hematopoietic stem cell transplantation (allo-HSCT) is an established curative treatment for pediatric patients with SAA. Busulfan, a key component of conditioning regimens, has a narrow therapeutic index and marked interindividual pharmacokinetic variability. However, the impact of different first-dose busulfan exposure levels on long-term outcomes, particularly immune reconstitution, remains unclear in pediatric patients receiving a 2-day conditioning regimen. We evaluated the clinical significance of busulfan exposure stratification in pediatric patients with SAA undergoing allo-HSCT.

**Methods:**

This retrospective study included 40 pediatric patients with SAA who underwent allo-HSCT. Patients were stratified into a low-exposure group (first-dose area under the concentration-time curve [AUC_0-t_] <900 μmol min/L, n = 24, 1 mg h/L ≈ 243.6 μmol min/L) and a standard-exposure group (AUC_0-t_ ≥900 μmol min/L, n = 16). Engraftment kinetics, transplant-related complications, and survival outcomes were compared between the groups. Longitudinal immune reconstitution (T-cell, NK-cell, B-cell counts, and immunoglobulin levels) was analyzed using linear mixed-effects models with Holm adjustment for multiple testing.

**Results:**

No significant differences were observed between the groups in neutrophil or platelet engraftment, incidence of graft-versus-host disease, hemorrhagic cystitis, engraftment syndrome, or short-term survival outcomes. All nine immune outcomes changed significantly over the first 12 months after transplantation, documenting pronounced temporal immune reconstitution (All adjusted *P* < 0.001 except IgM: *P* = 0.002). However, exposure-related differences in these trajectories were not confirmed after Holm adjustment (all adjusted *P* > 0.05).

**Conclusion:**

First-dose busulfan AUC_0-t_ < 900 μmol min/L (3.7 mg h/L) was not associated with impaired engraftment or increased early toxicity in pediatric patients with SAA undergoing allo-HSCT. The predominant longitudinal immune finding was time-dependent immune reconstitution, without confirmatory evidence that the two exposure groups followed different trajectories. These findings are limited by the modest sample size and retrospective design; therefore, prospective validation is needed to inform dosing strategies.

## 1 Introduction

Severe aplastic anemia (SAA) is a life-threatening condition characterized by bone marrow failure. For suitable pediatric patients, allogeneic hematopoietic stem cell transplantation (allo-HSCT) has demonstrated substantial efficacy and is an established treatment (Guarina et al., 2024). The conditioning regimen is a key component of transplantation, contributing to the eradication of aberrant immunity, suppression of host rejection, and creation of space for donor stem cell engraftment. Its intensity and safety are closely associated with transplant success and long-term patient survival. Busulfan is a bifunctional alkylating agent with complex pharmacokinetics and marked interindividual variability, particularly in pediatric patients. Busulfan-based combination regimens are widely used in pediatric SAA transplantation; however, busulfan has a narrow therapeutic index, and both under- and overexposure can adversely affect transplant outcomes (Shao et al., 2022). Busulfan exposure, typically measured by the area under the concentration-time curve (AUC_0-t_), correlates with efficacy and toxicity. Therefore, therapeutic drug monitoring (TDM) and subsequent dose adjustment based on pharmacokinetic results have become standard strategies for optimizing busulfan therapy and reducing adverse reactions (Bartelink et al., 2016; Domingos et al., 2024; Shimano et al., 2024).

International guidelines and the drug label recommend a target AUC_0-t_ range of 900–1,350 or 900–1,500 μmol min/L (1 mg h/L ≈ 243.6 μmol min/L) per dose for intravenous busulfan in pediatric patients. These recommendations are primarily based on data from patients with malignant hematological diseases receiving a 4-day regimen, in whom the treatment goal is myeloablation and antitumor activity (Domingos et al., 2024; Pfizer Inc., 2023). For pediatric SAA, the primary conditioning objective is to achieve sufficient immunosuppression to support donor engraftment, rather than the eradication of malignant clones. Consequently, whether AUC_0-t_ targets established for malignant diseases are directly applicable to pediatric patients with SAA remains uncertain, and the optimal busulfan exposure target in pediatric SAA has yet to be defined (Shimano et al., 2024; Wirk, 2024).

Given the goal of ensuring effective engraftment and disease control, relatively low busulfan exposure may be sufficient to achieve therapeutic objectives while potentially reducing treatment-related toxicities and improving quality of life (Güngör et al., 2014). Furthermore, in pediatric SAA transplantation, busulfan is often administered as a short 2-day consecutive course, which differs significantly from the 4-day regimen commonly used for malignant diseases. By the second day of administration, near steady-state concentrations are achieved, limiting the opportunity for timely dose adjustments based on pharmacokinetic monitoring and thereby highlighting the importance of first-dose pharmacokinetic results. In addition, the effect of busulfan exposure intensity on post-transplant immune reconstitution in pediatric patients with SAA remains poorly understood.

This retrospective study was designed to address these gaps by evaluating the clinical significance of first-dose busulfan exposure stratification in pediatric patients with SAA undergoing allo-HSCT. Patients were stratified according to first-dose busulfan AUC_0-t_ into low- and standard-exposure groups, and engraftment, transplant-related complications, survival outcomes, and long-term immune reconstitution were compared.

## 2 Materials and methods

### 2.1 Study design and population

Forty pediatric patients who underwent allo-HSCT for aplastic anemia at Hebei Children’s Hospital between October 2023 and October 2025 were enrolled in this study: 37 with SAA, 2 with non-severe AA, and 1 with SAA–paroxysmal nocturnal hemoglobinuria (PNH). All patients were diagnosed according to the Chinese guidelines for the diagnosis and management of aplastic anemia (Red Blood Cell Disease GroupChinese Society of Hematology Chinese Medical Association, 2022). Written informed consent was obtained from the legal guardians of all participants before transplantation. The study protocol was approved by the Institutional Ethics Committee of our hospital (Approval Number: 2023-12).

### 2.2 Treatment and blood sampling protocols

#### 2.2.1 Conditioning regimen and busulfan TDM

All patients were admitted to a laminar airflow isolation unit (Class 100). The conditioning regimen comprised busulfan (Bu)/cyclophosphamide (CTX)/fludarabine (Flu) + anti-thymocyte globulin (ATG), administered as follows: Bu 3.2–4.8 mg/kg/day for 2 days, CTX 60 mg/kg/day for 2 days, Flu 35 mg/m^2^∙day for 5 days, and ATG 10 mg/kg divided over 3 days. Pharmacokinetic parameters, including AUC_0-t_, were calculated for 19 patients who were enrolled before June 2024 using a conventional pharmacokinetic sampling strategy. For these patients, blood samples were collected before the first busulfan infusion and at 0, 15, 30, 60, 120, 135, 150, 180, 240, 300, and 360 min after the start of the infusion. For the remaining 21 patients enrolled after the establishment of the limited-sampling strategy in June 2024, the AUC_0-t_ was estimated using this alternative approach, with blood samples collected before the first busulfan infusion and at 60, 135, 240, and 360 min post-infusion. The choice of sampling method was not subject to selection bias but merely reflected a chronological change in the monitoring protocol over time. The agreement between AUC_0-t_ values determined using these two methods was validated in a previously published study (Jia et al., 2025). The limited-sampling strategy model employed in this study utilizes four time points (60, 135, 240, and 360 min post-infusion). In that validation cohort, the model demonstrated excellent predictive performance, achieving an adjusted *r* ^2^ of 0.965, an intraclass correlation coefficient of 0.983, and a low absolute prediction error of 1.89% ± 1.37%.

#### 2.2.2 Other treatments

Patients received oral posaconazole as antifungal prophylaxis from the start of the conditioning regimen. Intravenous ganciclovir was administered as antiviral prophylaxis until the day before stem cell infusion. During busulfan administration, patients older than 5 years received oral phenytoin sodium (5 mg/kg/day) for seizure prophylaxis. Alprostadil and ursodeoxycholic acid capsules were administered from the start of conditioning until 1 month post-transplantation to prevent hepatic veno-occlusive disease. For graft-versus-host disease (GVHD) prophylaxis, cyclosporine or tacrolimus combined with mycophenolate mofetil and short-course methotrexate was administered 24 h before stem cell infusion. GVHD prophylaxis consisted of cyclosporine A (CsA) in combination with short-course methotrexate (MTX). CsA was initiated on day −1 with a target trough concentration of 150–200 μg/L. MTX was administered intravenously at 15 mg/m^2^ on day +1 and 10 mg/m^2^ on days +3 and +6. Mycophenolate mofetil was optionally added to this core regimen, administered concurrently with CsA and discontinued following hematopoietic engraftment. Acyclovir was administered intravenously for 4 weeks after stem cell infusion to prevent Epstein-Barr virus (EBV) infection. Methylprednisolone was used as the first-line therapy for acute GVHD (aGVHD), and ruxolitinib was added if necessary. Severe or steroid-refractory aGVHD was treated with second-line therapies, such as CD25 monoclonal antibodies.

All patients received granulocyte colony-stimulating factor starting on day 4 post-transplantation, which was discontinued after neutrophil engraftment. Regular intravenous immunoglobulin support was provided following transplantation, and red blood cells, platelets, and other blood products were transfused as needed.

### 2.3 Evaluation of efficacy and safety

#### 2.3.1 Stem cell engraftment assessment

1. Neutrophil engraftment: Successful engraftment was defined as an absolute neutrophil count (ANC) > 0.5 × 10^9^/L for three consecutive days, with the first day considered the day of neutrophil engraftment.
2. Platelet engraftment: Successful engraftment was defined as a platelet count (PLT) > 20 × 10^9^/L for seven consecutive days without transfusion support, with the first day considered the day of platelet engraftment.

#### 2.3.2 Chimerism analysis

Chimerism was assessed using short tandem repeat polymerase chain reaction. Donor chimerism >95% was defined as complete donor chimerism, 5%–95% as mixed chimerism, and <5% as graft failure.

#### 2.3.3 Engraftment syndrome

Engraftment syndrome was defined as the occurrence of non-infectious fever, rash, diarrhea, and/or capillary leak syndrome during the early neutrophil recovery period.

#### 2.3.4 Diagnosis and grading of GVHD

Acute and chronic GVHD were diagnosed and graded according to established consensus criteria (Harris et al., 2016; Jagasia et al., 2015; Przepiorka et al., 1995).

#### 2.3.5 Hemorrhagic cystitis (HC) grading

The severity of post-transplant HC was graded using the internationally accepted four-grade classification: Grade I, microscopic hematuria; Grade II, gross hematuria without clots; Grade III, gross hematuria with clots; and Grade IV, gross hematuria with large clots causing urinary tract obstruction or requiring intervention or surgery. This grading system is described in the EBMT handbook, ASH guidelines, and several clinical studies. Graft failure was diagnosed according to internationally recognized criteria (Park et al., 2021).

#### 2.3.6 Primary outcome measures and follow-up

The evaluated outcomes included time to neutrophil and platelet engraftment, donor-recipient chimerism, transplant-related complications, incidence of GVHD, disease status, and survival. All patients were followed up through hospital records, outpatient visits, and telephone interviews. The follow-up endpoint was 20 March 2026, with a median follow-up duration of 660 days (range, 178–855 days). Overall survival (OS) was defined as the time from stem cell infusion to death from any cause or the last follow-up. GVHD-free survival (GFS) was defined as survival without any grade of GVHD (I-IV) or the aforementioned treatment failures. Immune reconstitution was monitored by assessing T-cell, B-cell, and natural killer (NK) cell counts, as well as immunoglobulin (IgM, IgG, and IgA) levels, at 1, 3, 6, 9, and 12 months post-transplantation.

### 2.4 Data statistics and analysis

#### 2.4.1 Pharmacokinetic parameters

Pharmacokinetic parameters were calculated using DAS 2.0 software (Chinese Pharmacological Society, Beijing, China) based on a non-compartmental model. The peak plasma concentration (C_max_) and time to reach C_max_ (T_max_) were obtained directly from the data. Other parameters, including AUC_0-t_, clearance, and apparent volume of distribution, were calculated using the software.

#### 2.4.2 Statistical analysis

Statistical analyses were performed using IBM SPSS Statistics, version 27.0.1 (IBM Corp., Armonk, NY, United States). Continuous variables were presented as mean ± standard deviation or median (range), as appropriate. Student’s t-test was used for normally distributed data and the Mann–Whitney *U* test was used for non-normally distributed data. Categorical variables were presented as numbers (%) and compared using the chi-square test. Survival outcomes were analyzed using the Kaplan-Meier method and the log-rank test. All tests were two-sided. Longitudinal immune-reconstitution outcomes were analyzed separately using linear mixed-effects models. Each model included exposure group (low exposure, first-dose AUC_0-t_ < 900 μmol min/L; standard exposure, first-dose AUC_0-t_ ≥ 900 μmol min/L), follow-up time (1, 3, 6, 9, and 12 months; categorical), and the group × time interaction as fixed effects. Cellular counts (CD3^+^, CD4^+^, CD8^+^, NK, and B cells) were analyzed after ln (x + 1) transformation, whereas the CD4/CD8 ratio and immunoglobulin concentrations were analyzed after ln(x) transformation. For each outcome, three candidate structures with the same fixed effects were compared: a random-intercept model with homogeneous residual variance, a random-intercept model with time-specific residual variances, and a random-intercept model with a centered linear-time random slope. Converged candidate structures were compared using maximum-likelihood Akaike information criterion (AIC) values, and the lowest-AIC structure was refitted using restricted maximum likelihood for inference. Denominator degrees of freedom were estimated using the Satterthwaite approximation. All available repeated observations were included without imputation under a missing-at-random assumption. Type III F tests evaluated the group, time, and group × time effects. Because overall temporal change and exposure-related trajectory divergence represented distinct hypotheses, the Holm procedure was applied separately to the nine time-effect tests and the nine group × time interaction tests. Holm-adjusted P < 0.05 was considered statistically significant within each test family. Group-by-time estimated marginal means and 95% confidence intervals were calculated on the model scale and back-transformed to the original measurement scale for graphical presentation. No pointwise between-group hypothesis tests were used for inferential conclusions.

## 3 Results

### 3.1 Baseline characteristics

The baseline characteristics of the study cohort are summarized in Table 1. Baseline characteristics are shown as n (%) or median (range). Since the groups were defined by exposure level rather than randomized, Table 1 reports descriptive statistics only; no formal between-group tests were performed. The median age was 9 years (range, 1–14), with a median body weight of 30.5 kg (range, 9.3–64.5), reflecting a heterogeneous pediatric cohort. Most patients received grafts from haploidentical parental donors (72.5%), and cord blood augmentation was used in 90% of patients. Based on busulfan pharmacokinetics, 24 patients (60.0%) were classified into the low-exposure group (AUC_0-t_ < 900 μmol min/L) and 16 (40.0%) into the standard-exposure group (AUC_0-t_ ≥ 900 μmol min/L). Groups were defined by pharmacokinetic exposure rather than randomized. Therefore, differences in donor composition across exposure strata are reported in Table 1. The median follow-up duration was 660 days (range, 178–855).

**TABLE 1:** Baseline characteristics of the study cohort ( N= 40).

Characteristic	Overall (N = 40)	Low-exposure group (N = 24)	Standard-exposure group (N = 16)
Demographics			
Age (years)	9 (1, 14)	9 (1, 14)	8 (1, 13)
≤10 years, n (%)	29 (72.5)	16 (66.7)	13 (81.3)
Sex, n (%)			
Male	26 (65.0)	18 (75.0)	8 (50.0)
Female	14 (35.0)	6 (25.0)	8 (50.0)
Weight (kg)	30.5 (9.3, 64.5)	37 (9.3, 64.5)	24.9 (10.0, 60.0)
ABO compatibility (between donor and recipient), n (%)			
ABO-matched	23 (57.5)	12 (50.0)	11 (68.8)
Minor ABO mismatch	9 (22.5)	7 (29.2)	2 (12.5)
Major ABO mismatch	8 (20.0)	5 (20.8)	3 (18.8)
Donor type, n (%)			
Haploidentical parental donor	29 (72.5)	14 (58.3)	15 (93.8)
Sibling donor	10 (25.0)	9 (37.5)	1 (6.3)
Unrelated donor	1 (2.5)	1 (4.2)	0
Cord blood augmentation, n (%)	36 (90.0)	20 (83.3)	16 (100.0)
HLA typing, n (%)			
10/10	4 (10.0)	3 (12.5)	1 (6.3)
6/10–9/10	11 (27.5)	8 (33.3)	3 (18.8)
5/10	25 (62.5)	12 (50.0)	13 (81.3)
Busulfan pharmacokinetic parameters			
C_max_ (μmol/L)	3.9 (2.9, 15.8)	3.8 (2.9, 4.5)	4.5 (3.3, 15.8)
AUC_0-t_ (μmol·min/L)	838.5 (618.6, 1,607.0)	792.2 (618.6, 892.6)	977.6 (918.3, 1,607.0)
Hematopoietic engraftment			
Infused MNC (×10^8^/kg)	9.4 (5.02, 20.0)	9.0 (5.02, 13.5)	9.5 (5.9, 20.0)
Infused CD34^+^ cells (×10^6^/kg)	6.5 (3.5, 11.2)	5.8 (3.5, 11.2)	6.5 (4.8, 9.0)

Data are presented as n (%) or median (range). Between-group statistical comparisons were not performed for Table 1; values are descriptive. Cord blood augmentation: co-infusion of one unrelated cord blood unit with haploidentical parental stem cells. All stem cell grafts were obtained from peripheral blood.

C_max_, peak plasma concentration; AUC, area under the concentration-time curve; MNC, mononuclear cells.

### 3.2 Pharmacokinetic parameters

**FIGURE 1:**
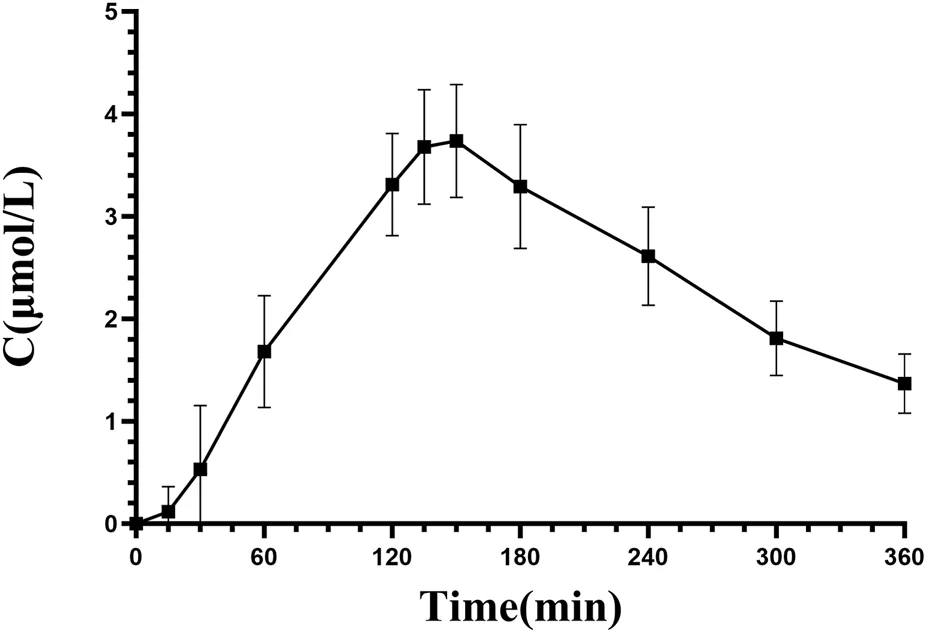
Mean plasma concentrations of intravenous busulfan in patients undergoing HSCT (N = 19). Mean plasma concentration–time profile of intravenous busulfan after the first conditioning dose in pediatric patients with severe aplastic anemia (n = 19) undergoing 2-day busulfan-based allo-HSCT. Blood samples were collected at 0, 15, 30, 60, 120, 135, 150, 180, 240, 300, and 360 min post-infusion for full pharmacokinetic sampling. Data points represent mean plasma busulfan concentration, with vertical error bars denoting SD.

The pharmacokinetic parameters of busulfan following the initial dose were analyzed in 19 pediatric patients. The mean area under the plasma concentration-time curve (AUC_0–t_) was 811.97 ± 90.34 μmol min/L. Other key parameters included a mean elimination half-life (t_1/2_) of 139.72 ± 40.68 min, time to peak concentration (T_max_) of 138.16 ± 8.03 min, peak concentration (C_max_) of 3.91 ± 0.49 μmol/L, and total body clearance of 0.17 ± 0.03 L/h/kg. The data demonstrated interindividual pharmacokinetic variability within the cohort. Detailed information is provided in Table 2 and Figure 1. Among nine patients with available day-2 pharmacokinetic data, the AUC_0-t_ measured on day 2 using a limited-sampling strategy was 1,292.33 ± 101.88 μmol min/L. The substantial day-2 AUC_0-t_ elevation in the nine assessed patients aligns with anticipated steady-state pharmacokinetics following multi-dose administration.

**TABLE 2:** Pharmacokinetic parameters of the first dose of busulfan (n = 19).

Parameters	Units	Mean ± SD
t_1/2_	min	139.72 ± 40.68
T_max_	min	138.16 ± 8.03
C_max_	μmol/L	3.91 ± 0.49
AUC_0-t_	μmol·min/L	811.97 ± 90.34
${\text{AUC}}_{0-\infty }$	μmol·min/L	1,092.56 ± 203.38
MRT_0-t_	min	186.50 ± 9.48
${\text{MRT}}_{0-\infty }$	min	282.75 ± 52.58
CL	L/h/kg	0.17 ± 0.03
Vd	L/kg	0.63 ± 0.13

t_1/2_, elimination half-life; T_max_, time to reach peak plasma concentration; C_max_, peak plasma concentration; AUC, area under the concentration-time curve; MRT, mean residence time; CL, clearance; V_d_, apparent volume of distribution.

### 3.3 Efficacy and safety outcomes

**FIGURE 2:**
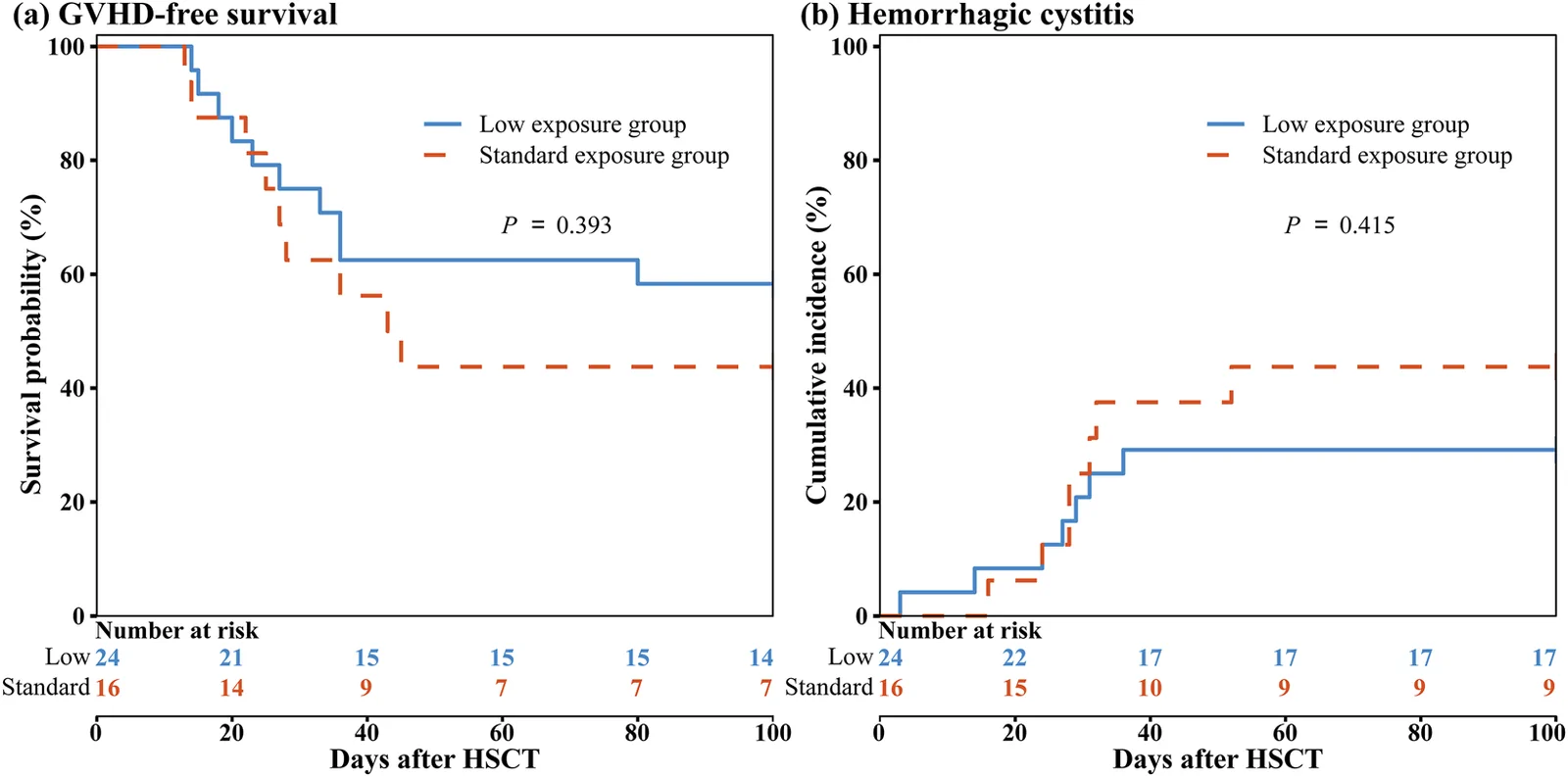
Comparison of GVHD-free survival and cumulative incidence of hemorrhagic cystitis within 100 days post-HSCT between the low- and standard-exposure groups. The low-exposure group was defined as a busulfan area under the concentration-time curve (AUC_0-t_) of <900 μmol min/L (solid blue line), and the standard-exposure group had an AUC_0-t_ ≥ 900 μmol min/L (dashed orange line). **(a)** Kaplan-Meier curves for GVHD-free survival; log-rank test, *P* = 0.393. **(b)** Cumulative incidence curves of hemorrhagic cystitis based on competing risk analysis, *P* = 0.415. Numbers at risk are presented at the bottom of each panel.

No significant differences were observed between the low- and standard-exposure groups regarding key post-transplant efficacy and safety outcomes. The time to neutrophil engraftment (12 vs. 11 days, *P =* 0.244), time to platelet engraftment (12 vs. 12 days, *P =* 0.141), and incidence of complications, including engraftment syndrome, hyperbilirubinemia, aGVHD, chronic GVHD (cGVHD), HC, and oral mucositis, were comparable between the two groups (all *P >* 0.05). No patients died or experienced primary graft failure (Table 3). Complete donor chimerism was achieved in all patients except one in the low-exposure group. One patient with VSAA in the low-exposure group (busulfan AUC_0-t_ = 889.68 μmol min/L) received combined sibling and cord blood grafts (MNC 8.9 × 10^8^/kg, CD34^+^ cells 5.22 × 10^6^/kg). Neutrophil and platelet engraftment were achieved on day +11 post-transplant. Grade II cutaneous aGVHD developed on day +20. Full donor chimerism (>98%) in bone marrow and peripheral T and B cells at 1 month was documented. The patient subsequently developed refractory persistent cytomegalovirus (CMV) infection, accompanied by progressive loss of donor chimerism down to 28.8% at day +68, severe marrow hypoplasia and secondary graft failure requiring a second allogeneic HSCT. Persistent CMV infection may have contributed to the development of secondary graft failure. Kaplan–Meier survival analysis demonstrated no significant differences in GVHD-free survival (GFS) or the incidence of HC between the low- and standard-exposure groups during the first 100 days post-transplantation (log-rank test: *P =* 0.393 and *P =* 0.415). The results are illustrated in Figure 2. None of the patients developed hepatic sinusoidal obstruction syndrome, seizures, severe liver injury, or severe pulmonary toxicity. Some patients experienced infections during treatment, all of which resolved after anti-infective therapy. No patient required admission to the intensive care unit.

**TABLE 3:** Comparison of post-transplant efficacy and safety between low and standard busulfan exposure groups.

Variables	Low-exposure group (n = 24)	Standard-exposure group (n = 16)	Statistics	*P*
Time to neutrophil engraftment (d)	12 (10, 15)	11 (10, 15)	U = 140.500	0.244
Time to platelet engraftment (d)	12 (10, 20)	12 (10, 18)	U = 260.500	0.141
Engraftment syndrome, n (%)	8 (33.3)	6 (37.5)	χ^2^ = 0.178	0.673
Primary graft failure	0 (0)	0 (0)	—	—
Secondary graft failure	1 (4.2)	0 (0)	Fisher’s exact	1.000
Hyperbilirubinemia	4 (16.7)	3 (18.8)	Fisher’s exact	1.000
aGVHD, n (%)	10 (41.7)	9 (56.3)	χ^2^ = 0.820	0.365
Absence of aGVHD	14 (58.3)	7 (43.8)	—	—
Grades 1, 2	5 (20.8)	7 (43.8)	—	—
Grades 3, 4	5 (20.8)	2 (12.5)	—	—
cGVHD, n (%)	7 (29.2)	4 (25.0)	Fisher’s exact	0.711
Hemorrhagic cystitis, n (%)	7 (29.2)	7 (43.8)	χ^2^ = 0.897	0.343
Without haemorrhagic cystitis, n (%)	17 (70.8)	9 (56.3)	—	—
Grades 1, 2	5 (20.8)	4 (25.0)	—	—
Grades 3, 4	2 (8.3)	3 (18.8)	—	—
Oral mucositis, n (%)	6 (25.0)	3 (18.8)	Fisher’s exact	0.717

Data are presented as n (%) or median (range). The low-exposure group was defined as a busulfan area under the concentration-time curve (AUC_0-t_) of <900 μmol min/L; the standard-exposure group had an AUC_0-t_ ≥ 900 μmol min/L. aGVHD, acute graft-versus-host disease; cGVHD, chronic graft-versus-host disease. All documented oral mucositis cases were grade 1–2 (CTCAE v5.0). All documented cGVHD, cases were characterized as mild-to-moderate and localized, with no progression to severe or extensive manifestations. No deaths occurred in either group. No primary graft failure, sinusoidal obstruction syndrome, seizures, severe liver injury, severe pulmonary toxicity, or ICU admission occurred.

### 3.4 Analysis of immune reconstitution

**FIGURE 3:**
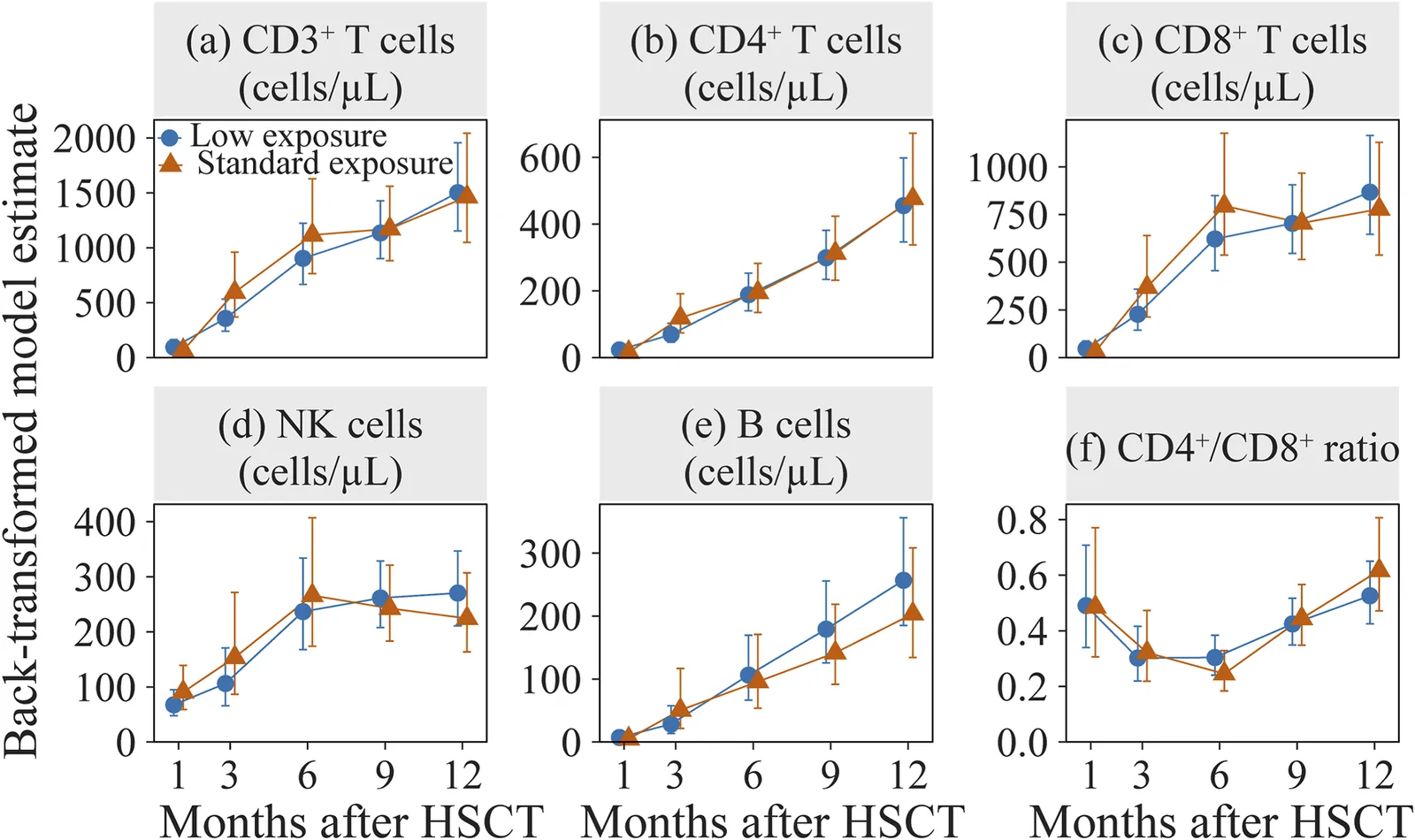
Trends in lymphocyte counts within 12 months post-HSCT in the low- and standard-exposure groups. **(a)** CD3^+^ T cells, **(b)** CD4^+^ T cells, **(c)** CD8^+^ T cells, **(d)** NK cells, **(e)** B cells, **(f)** CD4^+^/CD8^+^ ratio. Temporal profiles of cellular immune reconstitution during the first 12 months after HSCT, stratified by first-dose busulfan exposure. The low-exposure group was defined as AUC_0-t_ < 900 μmol min/L, and the standard-exposure group was defined as AUC_0-t_ ≥ 900 μmol min/L. Points represent back-transformed estimated marginal means and error bars represent 95% confidence intervals from outcome-specific linear mixed-effects models. Cell counts were modeled after ln (x + 1) transformation, whereas the CD4/CD8 ratio was modeled after ln(x) transformation. The numbers of evaluable patients at months 1, 3, 6, 9, and 12 were 24, 23, 21, 19, and 20 in the low-exposure group and 16, 15, 14, 13, and 12 in the standard-exposure group, respectively. All six cellular outcomes showed significant overall time effects after Holm adjustment (all adjusted P < 0.001), whereas none showed a significant group × time interaction after separate Holm adjustment (Table 4). No pointwise hypothesis tests are displayed.

**FIGURE 4:**
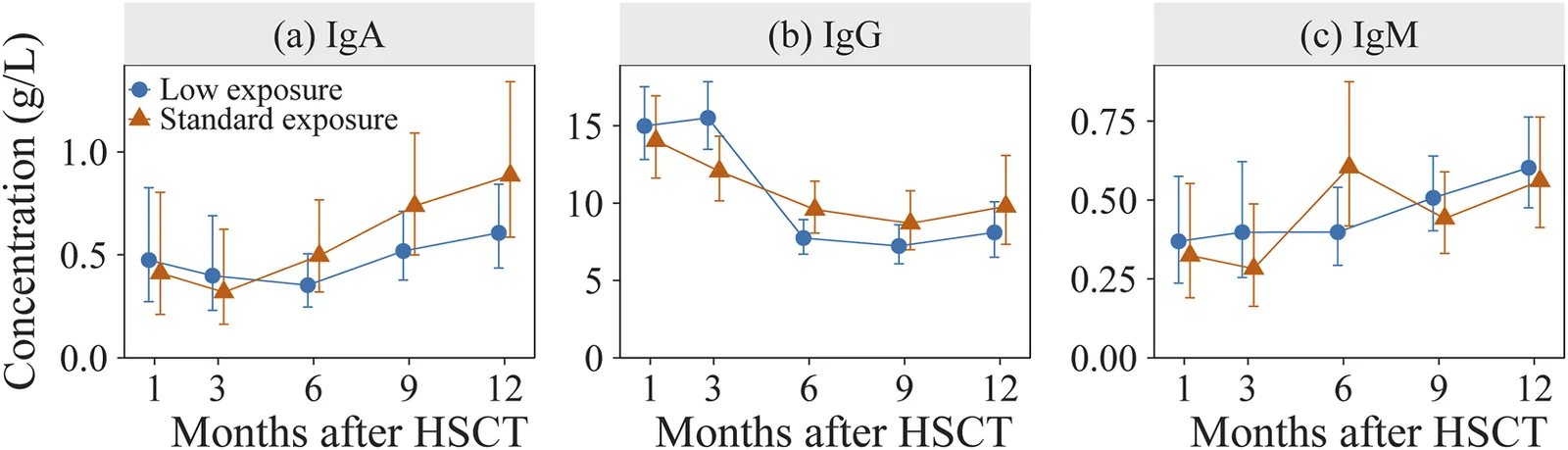
Trends in serum immunoglobulin (IgM, IgG, and IgA) levels within 12 months post-HSCT in the low- and standard-exposure groups. **(a)** IgA, **(b)** IgG, **(c)** IgM. Temporal profiles of serum immunoglobulin concentrations during the first 12 months after HSCT, stratified by first-dose busulfan exposure. The low-exposure group was defined as AUC_0-t_ < 900 μmol min/L, and the standard-exposure group was defined as AUC_0-t_ ≥ 900 μmol min/L. Points represent back-transformed estimated marginal means and error bars represent 95% confidence intervals from outcome-specific linear mixed-effects models fitted after ln(x) transformation. The numbers of evaluable patients at months 1, 3, 6, 9, and 12 were 24, 23, 23, 22, and 21 in the low-exposure group and 16, 16, 16, 14, and 11 in the standard-exposure group, respectively. Overall time effects remained significant after Holm adjustment for IgA and IgG (both adjusted P < 0.001) and IgM (adjusted P = 0.002), whereas none of the group × time interactions remained significant after separate Holm adjustment (Table 4). No pointwise hypothesis tests are displayed.

Linear mixed-effects models were fitted separately for the nine immune-reconstitution outcomes (Table 4). After Holm adjustment, all nine outcomes showed statistically significant overall time effects (adjusted P < 0.001 for eight outcomes and P = 0.002 for IgM). None of the nine group × time interactions was statistically significant after a separate Holm adjustment (all adjusted P ≥ 0.05). IgG yielded the smallest unadjusted interaction P value (F(4, 85.60) = 3.83, P = 0.007); however, this result did not remain statistically significant after Holm adjustment (P = 0.059) and was therefore interpreted as exploratory. Overall, the principal longitudinal finding was time-dependent immune reconstitution during follow-up. Thus, the predominant longitudinal finding was time-dependent immune reconstitution across follow-up, without confirmatory evidence that the two exposure groups followed different trajectories. Figures 3, 4 display the corresponding back-transformed estimated marginal means and 95% confidence intervals. Figures 3, 4 collectively illustrate the longitudinal immune reconstitution profiles based on linear mixed-effects models, encompassing both cellular immunity (T cells, NK cells, B cells, and their subsets) and humoral immunity (immunoglobulins). Over the 12-month follow-up period, both the low-exposure and standard-exposure groups exhibited clear trends in immune recovery. Specifically, absolute counts of T cells (CD3^+^, CD4^+^, CD8^+^) and NK cells progressively increased over time, while B cell counts steadily recovered. Concurrently, in terms of humoral immunity, serum levels of IgG, IgA, and IgM, after initial fluctuations, gradually rebounded or stabilized at certain levels during the later follow-up period. Overall, the trajectories of both cellular and humoral immune reconstitution showed substantial overlap between the two groups, with no significant inter-group differences observed.

**TABLE 4:** Longitudinal time effects and exposure-group × time interactions for immune-reconstitution outcomes.

Outcome	Valid observations	Time effect, F(df)	Time raw P	Time Holm P	Group × time, F(df)	Interaction raw P	Interaction Holm P
CD3^+^ T cells	177	F(4, 47.87) = 57.20	<0.001	<0.001	F(4, 47.87) = 1.38	0.257	1.000
CD4^+^ T cells	177	F(4, 38.31) = 88.28	<0.001	<0.001	F(4, 38.31) = 1.22	0.320	1.000
CD8^+^ T cells	177	F(4, 46.38) = 48.21	<0.001	<0.001	F(4, 46.38) = 1.55	0.203	1.000
NK cells	177	F(4, 57.29) = 26.28	<0.001	<0.001	F(4, 57.29) = 1.39	0.250	1.000
B cells	177	F(4, 48.71) = 62.27	<0.001	<0.001	F(4, 48.71) = 0.63	0.641	1.000
CD4^+^/CD8^+^ ratio	177	F(4, 51.68) = 23.89	<0.001	<0.001	F(4, 51.68) = 1.39	0.250	1.000
IgA	186	F(4, 53.82) = 12.65	<0.001	<0.001	F(4, 53.82) = 1.10	0.366	1.000
IgG	186	F(4, 85.60) = 22.10	<0.001	<0.001	F(4, 85.60) = 3.83	0.007	0.059
IgM	186	F(4, 50.24) = 5.09	0.002	0.002	F(4, 50.24) = 2.03	0.104	0.832

Values are Type III F tests from outcome-specific linear mixed-effects models with Satterthwaite denominator degrees of freedom. All models included exposure group, categorical follow-up time, and their interaction. Cell counts were analyzed after ln (x + 1) transformation; the CD4/CD8 ratio and immunoglobulin concentrations were analyzed after ln(x) transformation. Candidate structures were compared using maximum-likelihood AIC, and the selected model was refitted using restricted maximum likelihood. A random intercept with time-specific residual variances was selected for all outcomes except IgG, for which a random intercept plus centered linear-time random slope was selected. Holm adjustment was applied separately across the nine time-effect tests and the nine group × time interaction tests. All models included 40 participants. Overall group main effects were non-significant for all outcomes.

## 4 Discussion

Establishing an appropriate busulfan AUC_0-t_ range for pediatric patients with SAA undergoing allo-HSCT requires balancing efficacy and toxicity. Based on previous studies and consensus recommendations, determination of the optimal busulfan AUC_0-t_ target in pediatric patients with SAA requires consideration of the autoimmune pathophysiology of the disease rather than the myeloablative goals used in hematologic malignancies (Domingos et al., 2024; Shimano et al., 2024). Busulfan is an important conditioning agent with a relatively narrow therapeutic window and marked interindividual pharmacokinetic variability, particularly in pediatric patients (Marsit et al., 2020; Puangpetch et al., 2024; Schreib et al., 2023). For pediatric patients receiving busulfan-containing conditioning regimens for allo-HSCT, TDM on the first day of treatment may help achieve the optimal busulfan exposure target (Bognàr et al., 2023).

Most pharmacokinetic studies of busulfan in pediatric populations have enrolled patients with various primary diseases, predominantly malignancies, who received busulfan for four consecutive days. However, these studies did not specifically focus on pediatric patients with SAA, who typically receive busulfan over two consecutive days. For patients receiving the 2-day regimen, pharmacokinetic parameters obtained after the initial busulfan dose served as the primary reference. Although busulfan concentrations may approach steady state by the second day, by the time steady-state AUC_0-t_ data become available, drug administration is nearly complete, leaving limited opportunity for dose adjustment. This formed the primary rationale for this study, which focused on first-dose busulfan AUC_0-t_ in pediatric patients with SAA. The pharmacokinetic data in this study were consistent with the values reported by Cremers et al. (2002) (population estimates: clearance = 0.29 L/h/kg and apparent volume of distribution = 0.84 L/kg), which were also obtained following the initial dose. However, our study included only pharmacokinetic parameters from the initial busulfan dose and therefore did not reflect steady-state data. Although near-steady-state AUC_0-t_ data were derived​ from nine patients using the limited-sampling strategy, the sparse sampling time points precluded accurate calculation of pharmacokinetic parameters. From a basic pharmacological perspective, the steady-state AUC_0-t_ values derived​ from these patients were consistent with the expected pharmacokinetic disposition of busulfan *in vivo*.

In this study, no statistically significant between-group differences were detected in engraftment time, the measured transplant-related complications (including engraftment syndrome, acute and chronic GVHD, HC, and oral mucositis), or short-term GFS. These findings have important clinical implications. Unlike transplantation for malignant diseases, which emphasizes sufficient busulfan exposure to achieve antitumor effects, the primary goal of conditioning in SAA is to provide adequate immunosuppression to ensure stable donor stem cell engraftment rather than the eradication of malignant clones. Our results suggest that, within the context of the current intensive conditioning regimen containing fludarabine, cyclophosphamide, and potent immunosuppressive agents such as ATG, lower busulfan exposure (AUC_0-t_ < 900 μmol min/L) may be sufficient to achieve the myeloablative goal, without observed additional risks of graft failure, GVHD, or other transplant-related complications in this cohort. Because this retrospective study was small and was not designed as an equivalence or non-inferiority study, these findings cannot establish a safe threshold or the minimum effective busulfan exposure. Prospective confirmation is required before the observed association can inform a dosing threshold. These findings are consistent with the concept of exploring the minimal effective intensity of conditioning in non-malignant diseases to reduce long-term toxicity (Güngör et al., 2014; Satwani et al., 2008). Lower busulfan exposure may also reduce the risk of sinusoidal obstruction syndrome in pediatric patients with SAA (Gurlek Gokcebay et al., 2022).

Immune reconstitution after allo-HSCT is a complex and gradual process. Recovery of immune cells and reconstitution of the immune system are closely associated with post-transplant complications, such as infections and GVHD, which ultimately affect patient prognosis (Bhatt and Bednarski, 2020; Mellgren et al., 2019). Immune reconstitution is a key indicator of long-term post-transplant survival and is influenced by multiple factors. Consistent with previous findings (Wang et al., 2022), NK cells recovered most rapidly during immune reconstitution in pediatric patients with SAA after allo-HSCT, followed by T-cell recovery. In contrast, B-cell recovery was the slowest, with normalization occurring after approximately 1 year. CD8^+^ T cells recovered more rapidly than CD4^+^ T cells. IgA recovery was slower than IgM recovery. Because pediatric patients receive intermittent immunoglobulin infusions following allo-HSCT to prevent viral infections, measured IgG levels may not accurately reflect endogenous IgG production.

Busulfan exposure is an important factor influencing immune reconstitution after pediatric allo-HSCT. Appropriate exposure may promote recovery of T cells, B cells, and neutrophils, which may contribute to improved clinical outcomes, whereas excessive exposure may exacerbate immune injury, increase the risk of GVHD, and delay immune reconstitution (Bognàr et al., 2024; Feng et al., 2020; Yue et al., 2026). The significant time effects observed across all nine immune outcomes provide the clearest longitudinal immune finding of this study: cellular and humoral immune measures evolved substantially during the first post-transplant year. Together with the model-estimated trajectories shown in Figures 3, 4, these results support the clinical relevance of longitudinal immune monitoring. The results of this study demonstrate highly consistent trajectories of immune reconstitution between the low-exposure and standard-exposure regimens, spanning both cellular and humoral immunity. Neither the recovery patterns of T cells, NK cells, and B cells, nor the dynamic changes in the three major immunoglobulins, showed apparent delays or suppression attributable to reduced busulfan exposure. This holistic observation suggests that, within the context of the current intensive conditioning regimen containing fludarabine, cyclophosphamide, and ATG, lower busulfan exposure (AUC_0-t_ < 900 μmol min/L) may not exert a significant adverse effect on the overall immune reconstitution process. This finding implies the potential of the low-exposure strategy to ensure adequate immune recovery, offering preliminary theoretical support for optimizing conditioning regimens to mitigate drug toxicity. However, its long-term safety and efficacy require further validation through larger, prospective clinical trials.

This study has several limitations inherent in its retrospective, single-center design and modest sample size (n = 40). First, because exposure groups were defined by actual busulfan AUC_0-t_ rather than by randomization, baseline imbalances in donor type and HLA matching may have confounded between-group comparisons. Second, immune measurements were incomplete at later follow-up points; although the mixed-effects models included all available observations under a missing-at-random assumption, this approach cannot fully exclude bias from informative missingness. Third, exposure classification was based on first-dose AUC_0-t_, as day-2 pharmacokinetic data were available for only a small subset of patients. Finally, despite significant time effects after correction for multiple testing, the sample size provided limited precision for detecting exposure-related differences in longitudinal immune trajectories, and non-significant interactions should not be interpreted as evidence of equivalence. These findings therefore require confirmation in larger prospective cohorts.

In this retrospective cohort of pediatric patients with aplastic anemia receiving a 2-day busulfan-based conditioning regimen, a first-dose AUC_0-t_ < 900 μmol min/L was not associated with inferior engraftment, increased early toxicity, or worse short-term GFS compared with an AUC_0-t_ ≥ 900 μmol min/L. All nine immune outcomes changed significantly over the first 12 months after transplantation, documenting pronounced temporal immune reconstitution, whereas exposure-related differences in these trajectories were not confirmed after Holm adjustment. Day-2 measurements in a small subset suggest that steady-state AUC_0-t_ was significantly higher than the initial-dose AUC_0-t_, implying that increasing the initial dose may not be necessary; however, these data alone are insufficient to guide dosing decisions. Larger prospective studies are needed to compare exposure–outcome relationships across the exposure spectrum.

## Data Availability

The raw data supporting the conclusions of this article are not publicly available due to patient privacy and ethical restrictions but are available from the corresponding author upon reasonable request.
